# Technique de Blount dans le traitement des fractures supra condyliennes du coude chez l'enfant: à propos de 68 cas

**DOI:** 10.11604/pamj.2014.19.52.5299

**Published:** 2014-09-22

**Authors:** Aniss Chagou, Abdelkarim Rhanim, Rachid Zanati, Mohammed Kharmaz, Moulay Omar Lamrani, Mohammed Saleh Berrada, Moradh El Yaacoubi, Fouad Ettaybi

**Affiliations:** 1 Service de Chirurgie Orthopédique et Traumatologique CHU Ibn Sina de Rabat, Université Mohammed V, Rabat, Maroc; 2 Service des Urgences Chirurgicales Pédiatriques Hôpital d'enfant de Rabat, Université Mohammed V, Rabat, Maroc

**Keywords:** Humérus, fracture supra condyliennes, enfant, traitement orthopédique, humerus, supra condylar fracture, child, orthopedic treatment

## Abstract

La fracture de la palette humérale est la plus fréquente des fractures du coude de l'enfant. La méthode de BLOUNT, constitue une perspective thérapeutique longtemps connue. Elle consiste en une réduction sous contrôle scopique de la fracture et une contention en hyper flexion du coude. Notre série a porté sur l’étude de 68 cas de fractures supra condyliennes chez des enfants traités dans le service des urgences chirurgicales pédiatriques de l'hôpital d'enfant de Rabat entre janvier 2009 et janvier 2012. Nous comparons nos résultats avec les données de la littérature.

## Introduction

La fracture supra condylienne est une fracture métaphysaire extra articulaire de l'extrémité inférieure de l'humérus. C'est la plus fréquente des fractures du coude de l'enfant. Son pic de fréquence se situe entre 5 et 8 ans. Elle se produit au cours d'accidents de sport mais surtout de loisir et de vie courante [1]. La fracture en extension, où le fragment distal est basculé en arrière constitue quelque 95% des cas [1]. Deux classifications sont habituellement utilisées dans la littérature. La littérature française utilise la classification de Lagrange et Rigault [1]. La littérature anglo-saxonne fait référence à la classification de Gartland [2] La contention en flexion utilisant le périoste postérieur comme attelle interne maintenant la réduction est connue depuis longtemps. Le rapport princeps de Lagrange et Rigault [1] en 1962, a concouru à remplacer l'immobilisation en flexion plâtrée par la technique de Blount [3] Le but de ce travail est d’évaluer les résultats obtenus par la méthode de Blount, d'en expliquer précisément la technique rigoureuse en insistant sur l'importance fondamentale de l'apprentissage.

## Méthodes

**Figure 1:**
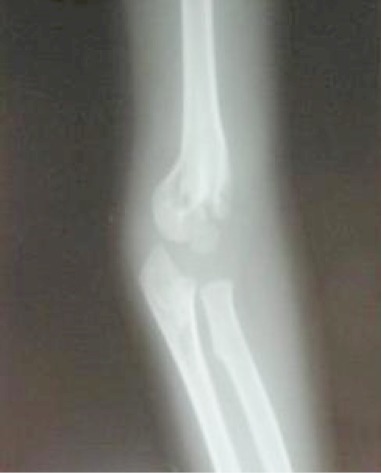
Radiographie de face et de profil du coude droit montrant une fracture supra condylienne stade II

**Figure 2:**
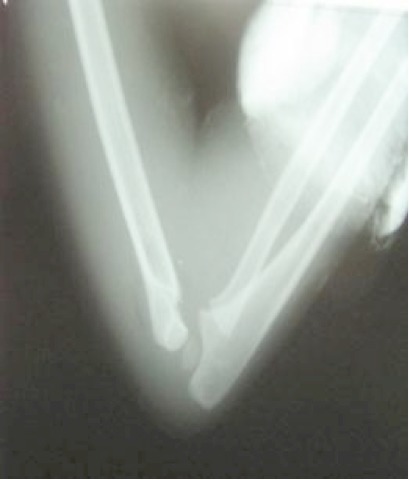
Radiographie de contrôle de profil après manœuvre de Blount montrant la bonne reduction

**Figure 3:**
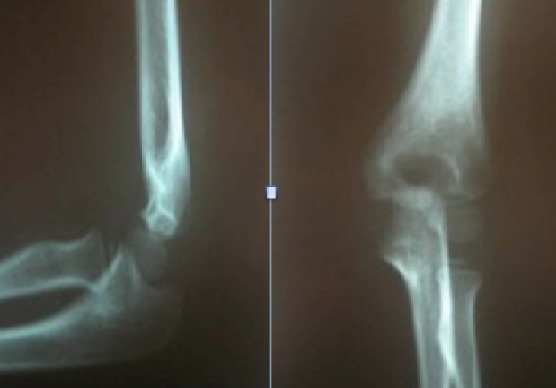
Radiographies chez le même enfant montrant une bonne consolidation osseuse

Notre travail s'attache à étudier les fractures de la palette humérale chez l'enfant traitées selon la méthode de Blount dans le service des urgences chirurgicales pédiatriques de Rabat durant une période de 3 ans entre janvier 2009 et janvier 2012, 68 dossiers cliniques ont été colligés. Tous nos patients ont été admis par le biais des urgences. L’âge était entre 2 et 13 ans. La moyenne d’âge globale est de 6,3 ans. On note une nette prédominance masculine avec un taux pour les garçons de 70% contre 30% pour les filles. Le côté gauche était atteint dans 53% des cas contre 47% pour le côté droit. La cause la plus fréquente des fractures de la palette humérale chez l'enfant est représentée par les accidents lors de l'activité sportive (79% des cas), suivi par les accidents domestiques (20%) puis les AVP (1%). Le mécanisme était chez tous nos patients une chute sur la main poignet et coude en extension. Le délai moyen entre le traumatisme et la consultation aux urgences est de 7,6 h avec un minimum de 1h30 et un maximum de 24h. La douleur du coude et l'impotence fonctionnelle du membre supérieur sont retrouvées chez tous nos patients. L’œdème est retrouvé dans 40 cas (59%), jugé minime dans 30 cas, modéré dans 8 cas et important dans 2 cas. Aucune lésion vasculo nerveuse n'a été retrouvée et aucune ouverture cutanée n'a été rapportée. Tous les patients ont bénéficié d'une radiographie du coude face et profil (Figure 1). L'analyse radiologique se base sur la classification de LAGRANGE ET RIGAULT [1]. Il s'agit d'un stade II dans 31 cas (45,5%), un stade III dans 33 cas soit 48,5% et un stade IV dans 4 cas soit 5,8%. La technique de Blount est pratiquée sous anesthésie générale au bloc opératoire. Après réalisation de la technique une radiographie de contrôle est faite au réveil de l'enfant (Figure 2). Les parents sont informés des complications immédiates de la technique nécessitant une consultation aux urgences, de même la nécessité de respecter le dispositif mis en place. Une radiographie de contrôle est réalisée à J8 afin de déceler un éventuel déplacement secondaire. La durée d'immobilisation était de 30 jours une radiographie de contrôle est faite en fin de traitement (Figure 3).

## Résultats

L'appréciation du résultat final nous a été possible par l’étude des données fournies par les consultations régulières des enfants traités par la technique de BLOUNT. Les résultats fonctionnels sont analysés selon les critères de LAGRANGE ET RIGAULT [1]: **Résultat parfait ou excellent:** un coude normal à tout point de vue, strictement identique au côté opposé. **Résultat bon:** coude dont l'utilisation fonctionnelle est normale mais au niveau duquel il persiste: Soit un léger déficit de la force musculaire; Soit un léger déficit de la mobilité inférieur à 20° ne flexion extension; Soit à une modification d'axe égal ou inférieur à 10° à la condition que ce déficit soit isolé; Soit à une déformation inesthétique même minime. **Résultats médiocre:** Soit un coude ne permettant pas une utilisation fonctionnelle normale; Soit un coude dont le déficit musculaire st important; Soit un déficit de mobilité supérieur à 20°; Soit une déviation d'axe supérieure à 10. **Résultat mauvais:** Soit un coude fonctionnellement mauvais; Soit un déficit important de la force musculaire; Soit un déficit de la mobilité supérieur à 50°; Soit une déviation d'axe supérieur à 20°; Soit des troubles paralytiques persistantes.

**Figure 4:**
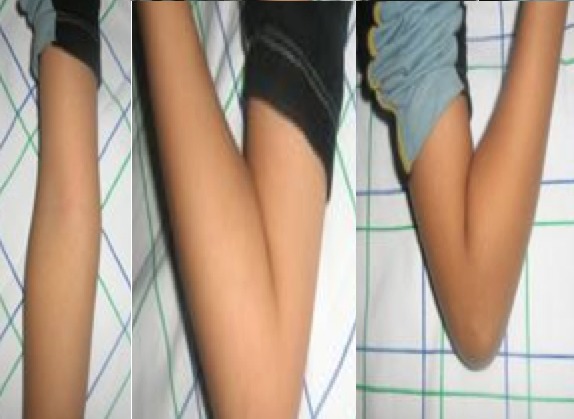
Résultat fonctionnel satisfaisant

Les résultats fonctionnels globaux sont résumés dans le Tableau 1. 49 cas (79%) de résultats excellents sont surtout obtenus pour les stades II et III. (Figure 4) 13 cas (19%) de bons résultats sont surtout observés pour les stades II et III. Le résultat médiocre pour le stade II a été noté chez un enfant dont les parents n'ont pas respecté le dispositif mis en place. Les résultats médiocres et mauvais notés pour le stade III sont des fractures vues tardivement avec un œdème important ayant gêné la réduction. Les résultats pour le stade IV sont aléatoires et dépendent de la qualité de la réduction initiale et celle de la surveillance.

**Tableau 1:** Résultats fonctionnels en fonction du stade de la fracture

	Résultats				
	Excellents	Bons	Médiocres	Mauvais	Total
Types					
Stade II	25 cas (36%)	5 cas (7%)	1 cas (1%)	0 cas	31 cas (45,5%)
Stade III	23 cas (33%)	7 cas (10%)	2 cas (2%)	1 cas (1%)	33 cas (48,5%)
Stade IV	1cas (1,3%)	1cas (1,3%)	1cas (1,3%)	1cas (1,3%)	4 cas (5,8%)
Total	49cas (72%)	13 cas (19%)	4 cas (5,8%)	2 cas (1,3%)	68 cas (100%)

Dans notre série, la réduction initiale était bonne chez 55 enfants (80%), satisfaisante chez 10 malades (14,7%) et non satisfaisante chez 3 malades (4,4%) chez qui un embrochage de type JUDET [4] a été pratiqué. Aucun syndrome de VOLKMAN n'a été noté chez les 68 malades traités selon la technique de BLOUNT. Trois déplacements secondaires ont été diagnostiqués lors de la radiographie de J8 (4%), ils ont été embrochés selon la technique de JUDET. Après un recul moyen de 1 an aucun cubitus varus n'a été noté chez nous patient et l'angle de Baumann moyen était de 78°.

## Discussion

La fracture supra condylienne est la lésion du coude la plus fréquente chez l'enfant et constitue environ 60% de toutes les fractures du coude [5]. Le pic de fréquence se situe entre l’âge de 5 à 7 ans [5] 6 à 9 ans dans notre série). La prédominance masculine est nette dans la plupart des séries de la littérature [5]. Il s'agit dans 95% des cas de mécanisme en extension. La douleur, l'impotence fonctionnelle et l’œdème représentent les signes cliniques les plus constants des fractures supra condyliennes. L'examen neuro vasculaire doit être systématique. L'atteinte du nerf interosseux antérieur est probablement la lésion neurologique la plus fréquente des fractures supra condyliennes, mais la plus méconnue [6]. Les lésions vasculaires surviennent dans environ 5% des cas, pratiquement exclusivement pour les fractures stade 4 [7]. Comme Clavert et al, nous n′avons retrouvé aucune complication vasculaire ou nerveuse [8].

Le bilan radiologique comprenant une radiographie du coude face et profil, permet la confirmation diagnostique, de classer la fracture selon la classification de LAGRANGE ET RIGAULT et de rechercher des fractures associées de l'avant-bras et du poignet. Le coude flottant survient le plus souvent après un traumatisme à haute énergie [9], la fracture supra condylienne est presque toujours en extension et celle de l'avant-bras siège souvent au quart distal, il s'agit même parfois d'une fracture décollement epiphysaire stage 2 de Salter et Harris [9].

La technique de Blount [3] comprend, une réduction urgente de la fracture afin d’éviter la survenu d’œdème et de troubles vasculo nerveux voir un syndrome de VLOKMAN [10] et une immobilisation du coude en hyper flexion de 120° pendant 4 semaines [11]. Clavert et Coll [8] insistent sur la nécessité d'une technique rigoureuse dans la confection du Blount qui permet d'avoir jusqu’à 90% de bons résultats. La pronation de l'avant-bras étant la position la plus stable pour tous les types de fractures supra condyliennes lorsque le coude est fléchi [12]. Cette technique ne concerne que les fractures en extension et son succès dépend en grande partie de l'intégrité du périoste postérieur, seul garant d'une mise en tension possible de ce périoste afin d'assurer un verrouillage en flexion maximale [13]. En effet, ce périoste est rompu une fois sur deux dans les fractures stade 4 ce qui explique les résultats aléatoires de la technique réalisée pour ce stade dans notre série. En plus le risque de complications ischémiques et de syndrome de Volkman est plus important [10].

Blount [3] a déconseillé sa technique dans les cas avec déficit vasculo nerveux et dans les fractures très déplacés avec un œdème important. Pour Damsin et Langlais [14] les contre-indications à la technique sont les fractures en flexion, les fractures instables et la présence de complications vasculaires. Par contre, l’œdème et la présence de complications nerveuses ne constituent pas une contre-indication absolue. En effet, Gupta et al. [15], en comparant deux groupes de patients, le premier vu dans les premières 12heures après le traumatisme et le second vu au-delà ce délai, n'ont pas trouvé de différence significative concernant le taux de réductions ouvertes et de complications péri opératoires entre les deux groupes. Dans notre série les résultats médiocres observés au stade III étaient des fractures vu tardivement avec un œdème important ayant gêné la réduction.

L’évaluation des résultats des enfants traités par cette technique comprend en plus de l’étude clinique et la fonction du coude, l’évaluation sur les radiographies de l'angle de Baumann, permettant de détecter une complication majeure de cette fracture: cubitus varus [16]. Cet angle, est formé par l'intersection entre l'axe de la diaphyse humérale et une ligne parallèle au cartilage du condyle latéral, il se mesure sur une incidence de face, sa valeur est de 75° plus ou moins 5°. Dans notre série nous n'avons noté aucun cas de cubitus varus l'angle de Baumann moyen était de 78°. CVA Kinkpé et al [17] dans sa série avait noté une tendance à la progression du cubitus varus entre les radiographies post réductionnels, les radiographies à l'union et au cours du suivi. Dowd et Hopcroft [18] et de Labelle et al [19] pensent que le cubitus varus n′entraîne pas de déficience fonctionnelle et que la perte est seulement esthétique. Etant donné que la déformation est seulement évidente en extension complète, ce qui implique que le patient a récupéré une mobilité complète [20]. Williamson et Cole [12] ont obtenu 95% d′excellents résultats avec la méthode Blount malgré 22,7% cubitus varus.

## Conclusion

Les fractures supra condyliennes sont les plus fréquentes de fractures du coude chez l'enfant. Elles prédominent chez les garçons ayant un âge compris entre 6 à 9 ans. Le mécanisme le plus fréquent est un mécanisme indirect. Le diagnostic sera suspecté par l'examen clinique qui doit rechercher systématiquement une complication immédiate. La confirmation est obtenue par la radiographie standard. Il s'agit d'une urgence thérapeutique. La technique de Blount permet d'obtenir de très bons résultats si on respecte la techniques les indications et les contre-indications. Une information des parents et leur collaboration est fondamentale.
